# Emerging Immune Functions of Non-Hematopoietic Stromal Cells

**DOI:** 10.3389/fimmu.2014.00437

**Published:** 2014-09-12

**Authors:** Christopher G. Mueller, Mark Christopher Coles

**Affiliations:** ^1^Centre National de la Recherche Scientifique, Strasbourg, France; ^2^University of York, York, UK

**Keywords:** stromal cells, immune cells, lymphoid tissue, inflammation, development

The proper function of the immune system is dependent on interactions between hematopoietic cells and non-hematopoietic stromal cells, which comprise the ensemble of tissue-forming cells (fibroblasts, endothelial cells, epithelial cells, nerve cells, etc.) that form the microenvironments in which immune responses occur (1, 2). This collection of cells is also referred to as the immune stroma. It creates a microenvironment in which the immune system operates, providing an architectural landscape for hematopoietic cell–cell interactions and molecular cues governing hematopoietic cell positioning, growth, and survival (3). It is the organization of stromal cell networks that imposes order on adaptive and innate immune cells and thus drives efficient immune responses. This interdependence of stromal cells of different lineages with all types of hematopoietic cells is therefore an integral part of primary (bone marrow, thymus), secondary (lymph nodes, mucosal-associated lymphoid tissue), and tertiary lymphoid tissue (that arise in response to chronic inflammation). Even in healthy tissue the type of stroma and its cellular activity influences its hematopoietic composition and may determine whether or not tertiary lymphoid structures form in case of unresolved inflammation, characteristic of many infectious, autoimmune, hematological malignancies, and inflammatory diseases (4).

One of the earliest motives that have fueled the study of immune stroma has been the interest in immune system development and the quest to define the action of a set of molecules required for primary and secondary lymphoid organ formation (5, 6). Some effects were radical leading to a total absence or lymphoid organs, whereas some were mild, resulting in the reduction of a subset of hematopoietic cells such as B cells owing to the absence of follicular dendritic cells. These early works have also laid one of the principle distinctions between stroma and hematopoietic cells, namely, the relative radioresistance of stroma. This observation has been fundamental to many experimental analyses aimed at deciphering the molecular makeup of stroma leading for instance to the discovery of lymphotoxin-sensitive stroma. This approach is now being displaced by the advent of mouse models allowing stroma and hematopoietic specific gene deletion and reporter gene expression. In addition, the development of techniques to analyze stroma by flow cytometry together with high resolution 4Dimensional microscopic techniques have helped to loosen the knot that has hampered fast-track discovery science.

The study of immune stroma and its interaction with hematopoietic cells is a natural progress in our quest as immunologists to understand how the immune system protects tissue and the vital organs that it forms or – in opposition – causes morbidity and mortality by tissue-directed immunopathology and thus translation to the clinic. Yet, it adds an additional layer of complexity to immunology by supplanting our knowledge of the already complex network of hematopoietic cell communications with that of tissue-forming cells. Communication systems between fibroblasts may be used by hematopoietic cells, or messages sent by stromal cells may be read by a subset of hematopoietic cells. Indeed, the importance of stress-released neuro-substances has a well-recognized, but yet insufficiently understood impact on our immune system. Although these imprints may be subtle, they are one of the multiple ways to affect the immune system in the long term and coupled to our increased life-span these subtle impacts will end up visible. Another contribution to the gain of importance of stroma in immunology has been the acceptance that tissue-forming cells turn-over and are often dependent on a somatic stem cells to renew dying cells. For immunologists, who embrace the notion of cell activation, proliferation, and cell precursors as an integral part of their thinking, this realization has contributed to the acceptation that stroma may be an important player in immune plasticity. Thus, stromal plasticity is likely to rise to become a critical issue in the understanding of immune homeostasis.

A future challenge will be to focus our investigation on the meaningful interaction between stroma and hematopoietic cells that determine the outcome of immune responses and not to be side tracked by observing the bystander effect of stroma cross-talk. The second challenge will be to translate these findings to pathology, in particular, tertiary lymphoid structure formation arising during chronic inflammation. Indeed, a better understanding of the molecular basis of stromal cell differentiation and function will provide potential new targets for therapeutic intervention in these diseases. Taken together, the study of non-hematopoietic stromal cells is an emerging theme in biology because (i) immunologists have realized that the immune system is strongly influenced by the tissue-forming cells, (ii) novel genetic mouse models and experimental tools are emerging to better address this issue, and (iii) by definition, it is an interdisciplinary field making the link between the hematopoietic cells and the tissue.

This collection of mini-reviews, perspectives, and original articles on stroma aims to attract the general attention of immunologists. It therefore includes different types of stromal cells: fibroblastic (FDCs, TRCs, and MRCs), blood, and lymphatic endothelial cells. Moreover, it focuses on primary, secondary, and tertiary lymphoid structures. Admittedly, this collection is far from complete, but it is a first brick in the building of our understating of stroma in the complex immunological landscape.

## Conflict of Interest Statement

The authors declare that the research was conducted in the absence of any commercial or financial relationships that could be construed as a potential conflict of interest.
